# An interplay between BRD4 and G9a regulates skeletal myogenesis

**DOI:** 10.3389/fcell.2022.978931

**Published:** 2022-09-07

**Authors:** Naidi Yang, Dipanwita Das, Shilpa Rani Shankar, Pierre-Alexis Goy, Ernesto Guccione, Reshma Taneja

**Affiliations:** ^1^ Department of Physiology, Healthy Longevity Translational Research Program, Yong Loo Lin School of Medicine, National University of Singapore, Singapore, Singapore; ^2^ Key Laboratory of Flexible Electronics (KLOFE) and Institute of Advanced Materials (IAM), Nanjing Tech University (NanjingTech), Nanjing, China; ^3^ Methyltransferases in Development and Disease Group, Institute of Molecular and Cell Biology, Agency for Science, Technology and Research (A*STAR), Singapore, Singapore; ^4^ Genome Institute of Singapore, Agency for Science, Technology and Research (A*STAR), Singapore, Singapore; ^5^ Department of Oncological Sciences, Tisch Cancer Institute, Icahn School of Medicine at Mount Sinai, New York, NY, United States

**Keywords:** differentiation, writer, reader, methylation, acetylation, muscle

## Abstract

Histone acetylation and methylation are epigenetic modifications that are dynamically regulated by chromatin modifiers to precisely regulate gene expression. However, the interplay by which histone modifications are synchronized to coordinate cellular differentiation is not fully understood. In this study, we demonstrate a relationship between BRD4, a reader of acetylation marks, and G9a, a writer of methylation marks in the regulation of myogenic differentiation. Using loss- and gain-of-function studies, as well as a pharmacological inhibition of its activity, we examined the mechanism by which BRD4 regulates myogenesis. Transcriptomic analysis using RNA sequencing revealed that a number of myogenic differentiation genes are downregulated in *Brd4*-depleted cells. Interestingly, some of these genes were upregulated upon G9a knockdown, indicating that BRD4 and G9a play opposing roles in the control of myogenic gene expression. Remarkably, the differentiation defect caused by *Brd4* knockdown was rescued by inhibition of G9a methyltransferase activity. These findings demonstrate that the absence of BRD4 results in the upregulation of G9a activity and consequently impaired myogenic differentiation. Collectively, our study identifies an interdependence between BRD4 and G9a for the precise control of transcriptional outputs to regulate myogenesis.

## Introduction

Skeletal myogenesis is precisely controlled by the myogenic regulatory factors (MRFs) Myf5, MyoD, and Myogenin (MyoG) and Mrf4 that are involved in various steps of proliferation and differentiation of muscle precursor cells (Sartorelli and Caretti, 2005; Bentzinger et al., 2012). In addition, multiple epigenetic modifiers also play important roles in regulating myogenesis (Saccone and Puri, 2010). Histone deacetylases (HDACs) and lysine methyltransferases (KMTs) mediate muscle gene repression, while acetyltransferases (KATs) promote muscle gene transcription and thus facilitate muscle differentiation (Saccone and Puri, 2010; Bharathy et al., 2013; Robinson and Dilworth, 2018; Karthik and Taneja, 2021). In myoblasts, several KMTs, such as Suv39h1 and G9a/GLP, mediate histone H3 lysine nine methylation (H3K9me), and Ezh2, which leads to histone H3 lysine 27 trimethylation (H3K27me3), represses differentiation genes (Zhang et al., 2002; Caretti et al., 2004; Mal, 2006; Ling et al., 2012; Ohno et al., 2013; Battisti et al., 2016; Choi et al., 2018). These KMTs are expressed in myoblasts and their downregulation is associated with activation of muscle gene expression and differentiation of satellite-cell derived myoblasts. In addition to histone modifications, nonhistone proteins such as MyoD and myocyte enhancer factor 2 D (MEF2D) are methylated by G9a resulting in repression of myogenesis (Ling et al., 2012; Choi et al., 2014; Jung et al., 2015). The KMT, Suv4-20h1 adds the H4K20me2 mark at the MyoD1 promoter and induces heterochromatin formation resulting in decreased MyoD1 expression in early activated muscle stem cells (Boonsanay et al., 2016). In addition, HDACs are also involved in the suppression of muscle differentiation in multiple ways, including deacetylation of histones or by direct interaction with transcription factors (Sincennes et al., 2016).

Upon induction of differentiation, HATs p300 and p300/CBP associated factor (P/CAF) activate MyoD, resulting in the transcriptional activation of myogenic genes *via* loci-specific histone acetylation mainly on H4K8 and H3K9 (Puri et al., 1997; Polesskaya et al., 2001; Roth et al., 2003; Khilji et al., 2018). p300 and P/CAF have also been shown to acetylate retinoblastoma tumor-suppressor protein (pRb) to mediate myogenesis (Nguyen et al., 2004). Arginine methyl transferases PRMT5 and CARM1, ATPase-dependent SWI/SNF chromatin-remodelling complexes and the MLL complex containing Ash2L interact with MRFs and MEF2 protein to initiate transcription of the target genes (Saccone and Puri, 2010).

Bromodomain-containing protein 4 (BRD4) belongs to the bromodomain and extraterminal domain (BET) family and contains two conserved N-terminal bromodomains (BD1 and BD2) and one extraterminal (ET) domain (Wu and Chiang, 2007). It was originally found to be associated with chromatin during mitosis and affect the G2/M transition (Dey et al., 2000) and later shown to enhance transcription *via* interaction with positive transcription elongation factor b (P-TEFb) to activate RNA Polymerase II (Pol II) (Jang et al., 2005; Yang et al., 2005). In addition, BRD4 is involved in transcriptional regulation *via* direct phosphorylation of Pol II, interaction with histone modifiers (such as Nsd3) or transcription factors (such as p53) (Wu et al., 2013; Shi and Vakoc, 2014). BRD4 also has intrinsic HAT activity. BRD4 mediates acetylation of the H3K122 residue, which leads to nucleosome eviction and thereby chromatin decompaction (Devaiah et al., 2016). BRD4 has been implicated both in physiological and pathological conditions (Di Micco et al., 2014; Liu et al., 2014; Korb et al., 2015; Wu et al., 2015; Kim et al., 2020; Lin et al., 2022; Paradise et al., 2022). In both human and mouse embryonic stem cells (ESCs), BRD4 positively regulates the expression of genes that are required for sustaining ESC identity (Di Micco et al., 2014; Liu et al., 2014; Wu et al., 2015). BRD4 has also been shown to be essential for hematopoietic stem cell development, cardiac function and neuronal function (Korb et al., 2015; Dey et al., 2019; Kannan-Sundhari et al., 2020; Kim et al., 2020). In addition, BRD4 is involved in inflammation by binding to acetylated NF-kB (Huang et al., 2009; Brown et al., 2014). In muscle cells, BRD4 is recruited by SMYD3 and involved in the regulation of *myostatin* and *c-Met* genes which mediate muscle atrophy (Proserpio et al., 2013). Consistently, inhibition of BRD4 prevents muscle damage both in Duchenne muscular dystrophy and cancer cachexia models (Segatto et al., 2017; Segatto et al., 2020). BRD4 has also been reported to regulate chondrocyte differentiation and endochondral ossification (Paradise et al., 2022), and plays a crucial role in myogenesis and adipogenesis (Lee et al., 2017; Roberts et al., 2017). However, the mechanisms by which BRD4 crosstalks with other chromatin modifiers to regulate myogenesis have not been elucidated.

In this study, we found that *Brd4* knockdown or pharmacological inhibition of its activity reduced cellular proliferation and skeletal muscle differentiation. RNA sequencing (RNA-seq) analysis of *Brd4* knockdown cells showed that it is strongly linked to muscle differentiation and structure. Since BRD4 promotes myogenesis, while the lysine methyltransferase G9a represses myogenesis, we examined the relationship between them by overlapping BRD4-regulated genes with G9a-regulated genes. Several genes important for myogenic differentiation such as *Myog* and *Myh1,* which were downregulated upon *Brd4* knockdown were upregulated by depletion of G9a. Consistently, upon induction of differentiation, BRD4 enrichment was increased on muscle differentiation genes such as *Myog* and *Myh1,* whereas G9a occupancy was reduced. Moreover, the block of myogenesis in siBrd4 cells was rescued by inhibition of G9a activity with UNC0642, demonstrating that BRD4 promotes myogenesis by inhibiting G9a. Consistent with these findings, treatment with UNC0642 led to an enrichment of the H3K9ac mark and BRD4 occupancy and a decrease in the H3K9me2 mark at the *Myog* promoter. Conversely, treatment with JQ1, which inhibits BRD4 activity, led to a decrease in H3K9ac and BRD4 occupancy and an increase in G9a and H3K9me2 marks at the *Myog* promoter. Altogether, the data suggest that the loss of myogenic differentiation in the absence of BRD4 is due to enhanced G9a activity and crosstalk between these two chromatin modifiers to regulate muscle differentiation.

## Materials and methods

### Cell culture, differentiation and proliferation assays

C2C12 cells were cultured in high glucose Dulbecco’s modified Eagle’s medium (DMEM, Sigma Aldrich) supplemented with 20% fetal bovine serum (FBS, HyClone). Primary mouse myoblasts were isolated as described previously (Sun et al., 2007). Cells were cultured in F-10 medium supplemented with 20% FBS and 5 ng/ml basic fibroblast growth factor (bFGF, Thermo Fisher Scientific) and plated on collagen-coated dishes. Phoenix cells were cultured in high glucose DMEM supplemented with 10% FBS. For differentiation, C2C12 cells or primary mouse myoblasts at 80%–90% confluency were cultured in differentiation medium (either in DMEM or F-10 medium) supplemented with 2% horse serum (Thermo Fisher Scientific). Differentiation was assessed by immunofluorescence as described previously (Ling et al., 2012). Briefly, cells cultured on sterile cover slips placed in 35 mm dishes were fixed with 4% paraformaldehyde for 15 min at room temperature, permeabilized and blocked using 10% horse serum in PBS containing 0.1% Triton X-100 for 45 min. Cells were then incubated with anti-MHC antibody (M4276, Sigma, 1:200 dilution) in blocking solution (PBS with 10% horse serum) for 1 h (hr) followed by secondary antibody tagged with fluorophore (Invitrogen). The cells were mounted using mounting agent (Vectashield) with DAPI. The images were captured by an Olympus (DP72) microscope. The myogenic index was calculated by quantifying the ratio of nuclei in myosin heavy chain-positive myotubes (more than three nuclei) to the total nuclei. More than 1,000 nuclei from five different fields were counted. Proliferation was measured by BrdU incorporation assays (Azmi et al., 2004). Briefly, cells were pulsed with 10 µM BrdU for 30 min and then fixed and stained with anti-BrdU antibody according to the manufacturer’s protocol (Sigma).

### Quantitative RT-PCR

Total RNA was extracted using TRIzol (Invitrogen), and complementary DNA (cDNA) was synthesized using an iScript cDNA Synthesis Kit (Bio-Rad). qRT-PCR was performed using a Lightcycler 480 SYBR Green one Master Kit (Bio-Rad). Primer sequences for *Myog* (Ling et al., 2012), *Myh1* (Choi et al., 2018), Cyclin D1 and Gapdh (Wang et al., 2012) have been described previously. Primers for *Brd4* were as follows: forward (Fw) 5′-CCA​TGG​ACA​TGA​GCA​CAA​TC-3′ and *Brd4* reverse (Rv) 5′-TGG​AGA​ACA​TCA​ATC​GGA​CA-3’; Dhrs7C (Fw) 5′-CCC​TGG​AGC​TTG​ACA​AAA​AGA-3′ and (Rv) 5′-GTT​CAC​TAA​CAC​AAT​CTG​GCC​T-3′.

### siRNA knockdown, plasmids, and retroviral infection

Cells were transfected with scrambled siRNA (Dharmacon, ONTARGETplus, Non-Targeting Pool) or *Brd4* siRNA (SC, sc141740) using Lipofectamine RNAiMAX (Invitrogen) according to the manufacturer’s instructions. After 48 h, cells were collected for analysis at Day 0 (D0) or shifted to differentiation medium. The pBabe-Brd4 plasmid was generated by inserting *Brd4* PCR products into the pBabe plasmid. To generate stable cell lines, retroviral infection of pBabe, pBabe-G9a or pBabe-Brd4 was performed as described previously (Rao et al., 2016). Briefly, plasmids were first transfected into Phoenix cells using CaCl_2,_ and the virus was then collected after 48 and 72 h C2C12 cells were then infected with the virus for 24 h and subjected to puromycin selection.

### Immunoblotting

Protein lysates for western blotting were harvested in Laemmli lysis buffer (62.5 mM Tris-HCl, pH 6.8, 20% glycerol, 2% SDS, 2 mM DTT) with proteinase inhibitor freshly added. The following primary antibodies were used for immunoblotting: anti-BRD4 (Bethyl, A301-985-50, dilution 1:5,000), anti-Myogenin (Santa Cruz Biotechnology, sc-576, dilution 1; 500), anti-Troponin-T (Sigma, T6277, dilution 1:2000), anti-β-actin (Sigma, A1978, dilution 1: 10,000), anti-G9a (Cell Signaling, 3306S, dilution 1: 300), anti-H3K9ac (Abcam, ab4441, dilution 1:1,000), and normal rabbit IgG (Santa Cruz Biotech, sc-2027).

### Immunostaining of mouse embryo sections

Sagittal paraffin sections of mouse embryos (Zymagen MP-104-008) at different stages of development (E12, E14, and E16 days) were analysed by IHC using an anti-BRD4 antibody (Bethyl, dilution 1:2000). Sections were incubated overnight at 4°C with anti-BRD4 antibody using Dako REAL EnVision-HRP, Rabbit-Mouse kit (Dako, Denmark). A negative control was performed using secondary antibody only. The sections were then counterstained with hematoxylin (Sigma-Aldrich). After dehydration, slides were mounted with DPX (Sigma-Aldrich) and imaged using an Olympus slide scanner (Olympus, Tokyo, Japan). Thereafter, an Aperio image scope viewer (Aperio, Vista, CA) was used to photograph the section.

### Chromatin immunoprecipitation

ChIP assays were performed as described previously (Ling et al., 2012). Briefly, 10^6^ cells were fixed in 1% formaldehyde for 10 min at 37°C and then collected and lysed in 1% SDS lysis buffer. Cells were sonicated using Bioruptor plus (Diagenode, Liege, Belgium). ChIP assays were performed according to the kit protocol (Millipore). Immunoprecipitates were reverse crosslinked, and DNA was extracted using phenol–chloroform–isoamyl alcohol (Sigma). qPCR was carried out in triplicate, and DNA isolated from 10% input was used as a control. The antibodies used for ChIP assays were as follows: anti-BRD4 (Bethly, A301-985-50), anti-G9a (ab40542), anti-H3K9ac (ab4441) and anti-H3K9me2 (ab1220) were from Abcam. Primers used for ChIP assays: Myogenin promoter (Fw): 5′-TGG​CTA​TAT​TTA​TCT​CTG​GGT​TCA​TG-3′ and (Rv): 5′-GCT​CCC​GCA​GCC​CCT-3’; Myh1 promoter (Fw): 5′- CACCCAAGCCGG GAGAAACAGCC-3′ and (Rv): 5′-GAGGAAGGACAGGACA GAGGCACC-3’; CyclinD1Ebox (Fw): 5′-GAG​AGC​TTA​GGG​CTC​GTC​TG-3′ and CyclinD1Ebox (Rv): 5′-TGG​GTG​CGT​TTC​CGA​GTA​C-3’.

### RNA-seq and data analysis

RNA-seq was performed with three biological replicates of scramble control and siBrd4 cells. 48 h after siRNA transfection, RNA was isolated from cells in growth medium (day 0) and after 1 day in differentiation medium (day 1). RNA was cleaned using an RNeasy MiniElute Cleanup Kit (Qiagen), and RNA quality was checked using a Bioanalyzer (Agilent Technologies). A TruSeq Stranded mRNA kit (Illumina) was used for construction of the mRNA library. The samples were sequenced on a NextSeq high output of a single read (1 × 76 bp). The analysis was performed as described previously (Tabaglio et al., 2018). At least 70 million 76-bp-long single-end reads were mapped to the mm9 version of the mouse genome per replicate using STAR 2.4.2a (Dobin et al., 2013) and differential expression analysis was performed using Cuffdiff 2.2.1 (Trapnell et al., 2012). Gene enrichment was performed using Metascape (Zhou et al., 2019) and GSEA (Subramanian et al., 2005). The *Brd4* RNA-seq data have been deposited in GEO under the accession number GSE141777. The G9a microarray data are available as GSE70039.

### Statistical analysis

Significance between two samples was calculated using two-tailed *t* test. Significance of more than three samples was calculated by one-way ANOVA with Tukey’s multiple comparisons test and multiple groups of two factors were compared by two-way ANOVA with Sidak’s multiple comparisons test using GraphPad Prism 8. *p* values of <0.05 were considered to be statistically significant (*p < 0.05, **p < 0.01, ***p < 0.001, ****p < 0.0001). Error bars represent the mean ± standard deviation (SD) of at least three independent experiments.

## Results

### Bromodomain-containing protein 4 expression declines during muscle differentiation

**FIGURE 1:**
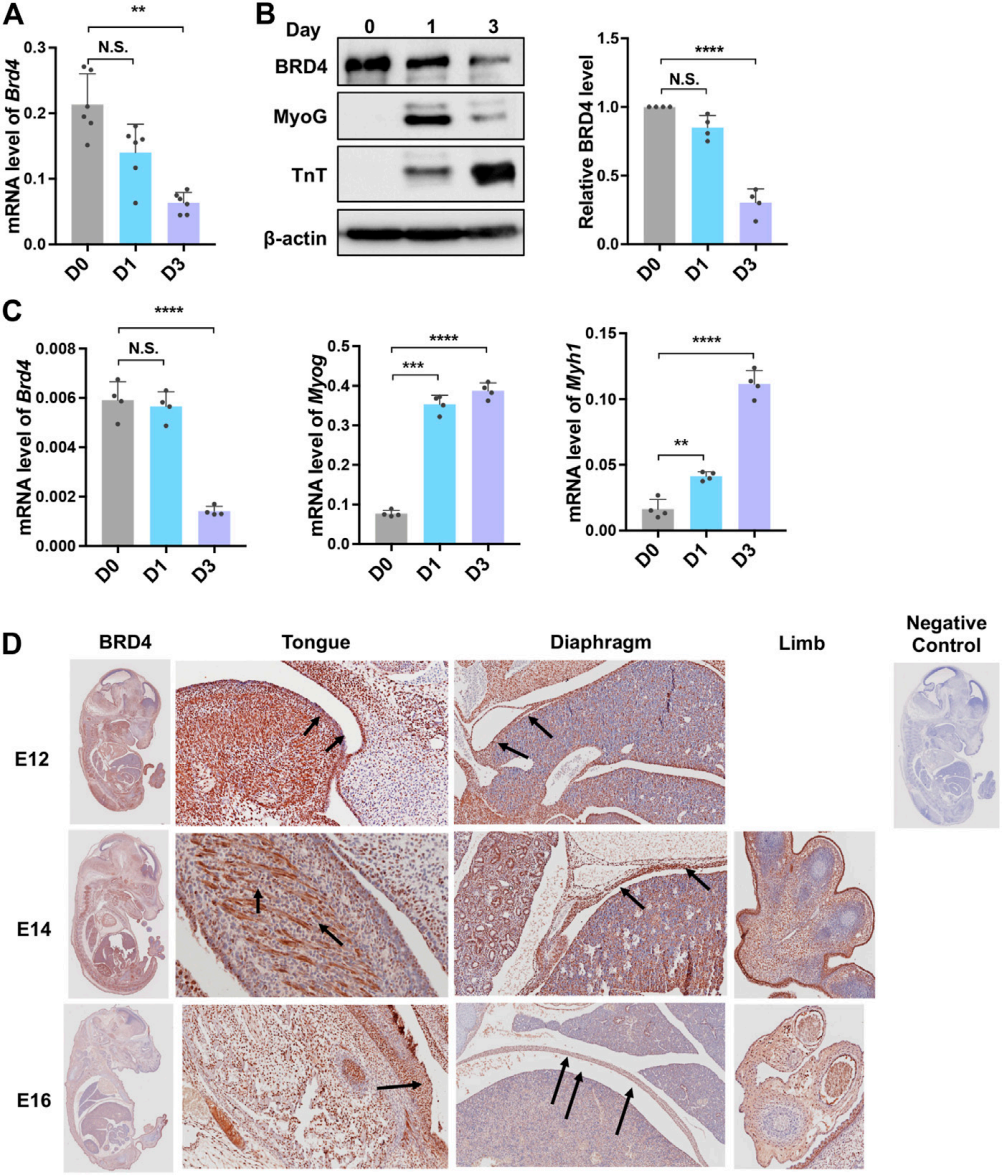
*Brd4* expression during myogenesis. **(A)** *Brd4* expression was analysed during myogenic differentiation of C2C12 cells. RNA isolated from undifferentiated cells (D0) and upon induction of differentiation (D1 and D3, respectively) was analysed by qRT-PCR, *n* = 6 independent experiments. **(B)** The expression of BRD4 and the myogenic differentiation markers MyoG and TnT was analysed by western blotting at D0, D1 and D3. β-actin was used as an internal control. BRD4 protein level from four independent experiments were analysed (normalized to β-actin and the relative protein level compared to D0) **(C)** *Brd4, MyoG* and *Myh1* expression was analysed in undifferentiated (D0) and differentiating (D1 and D3) primary mouse myoblasts by qRT-PCR. The data are the average from four independent experiments. **(D)** BRD4 IHC using sagittal sections from mouse embryos at E12, E14, and E16. Arrows indicate BRD4 staining. The negative control shows a section that was stained with secondary antibody only. Error bars indicate the mean ± SD. (**p < 0.01, ***p < 0.001, ***p < 0.0001, N.S. > 0.5, one way ANOVA with Tukey’s multiple comparisons test).

We first analysed BRD4 expression in C2C12 cells during myogenesis. *Brd4* mRNA levels slowly decreased during differentiation from day 0 to day 3 (Figure 1A). Consistently, the protein also declined upon differentiation and was inversely correlated with two well established differentiation markers, MyoG, which marks early differentiation, and TroponinT (TnT), which marks late differentiation (Figure 1B). A similar pattern was seen in primary myoblasts (Figure 1C). To further check its expression during embryonic development, we performed immunohistochemical staining on mouse embryo sections at E12, E14, and E16 using an anti-BRD4 antibody. BRD4 was prominently expressed in the developing muscles of the diaphragm, limb and tongue (Figure 1D).

### Loss or inhibition of bromodomain-containing protein 4 reduces muscle cell proliferation and differentiation

**FIGURE 2:**
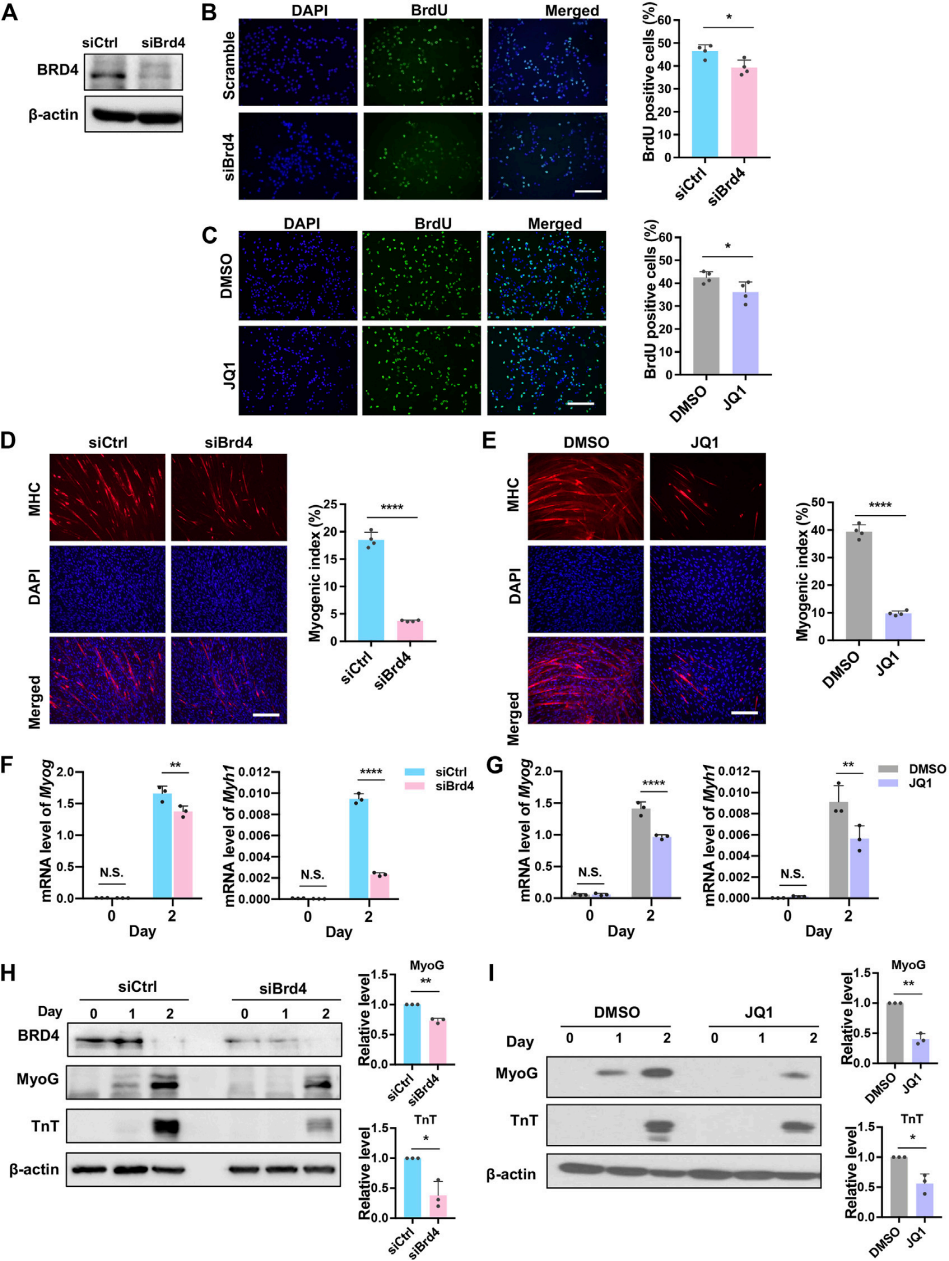
BRD4 loss reduces myoblast proliferation and myogenic differentiation. **(A)** C2C12 cells were transfected with either control siRNA (siCtrl) or *Brd4* siRNA (siBrd4) for 48 h. The siRNA-mediated *Brd4* knockdown efficiency was checked by western blotting. **(B)** siCtrl and siBrd4 cells were pulsed with BrdU and stained with anti-BrdU antibody (green). Nuclei were stained with DAPI (blue). Scale bar, 100 µm. The percentage of BrdU^+^ cells quantified by counting at least 1,000 cells (*n* = 4). **(C)** C2C12 cells were treated with 50 nM JQ1 for 24 h, pulsed with BrdU, and stained with an anti-BrdU antibody. Scale bar, 100 µm. The percentage of BrdU^+^ cells was quantified by counting at least 1,000 cells (*n* = 4). **(D)** siCtrl and siBrd4 cells were subjected to differentiation assays for 2 days and stained with MHC antibody (red) for detection of tube formation. Scale bar, 100 µm. Nuclei were stained with DAPI (blue). The bar graph shows the myogenic index that was quantified by the ratio of MHC^+^ cells to the total number of nuclei in the field. At least 1,000 nuclei from five fields of view were counted (*n* = 4). **(E)** C2C12 cells were pretreated with 50 nM JQ1 in growth medium and then differentiated with or without JQ1 for 2 days. Scale bar, 100 µm. MHC staining was performed and the myogenic index was quantified as described above (*n* = 4). **(F)** The mRNA levels of *Myog* and *Myh1* at D0 and D2 were measured by qRT-PCR in siCtrl and siBrd4 cells (*n* = 3). **(G)** C2C12 cells were pretreated with 50 nM JQ1 in growth medium and then differentiated with or without JQ1 for 2 days. The mRNA levels of *Myog* and *Myh1* were quantified by qRT-PCR (*n* = 3). **(H)** MyoG and TnT expression was determined by western blotting at D0, D1, and D2 in siCtrl and siBrd4 cells. β-actin was used as a loading control. To quantify the western blot signals, D2 MyoG and TnT expression at D2 were normalized to b-actin. The siCtrl (D2) was given a value of one and the relative expression in control and siBrd4 cells is shown in the bar graph. **(I)** C2C12 cells were treated as in **(G)**, and MyoG and TnT protein levels at D2 were detected by western blot. The bar graph shows the relative protein level as quantified above (*n* = 3). Error bars indicate the mean ± SD. (*p < 0.05, **p < 0.01, ****p < 0.0001, two-tailed *t* test was performed for Figures 2B–E and Figures 2H,I, two-way ANOVA was performed for Figures 2F,G).

Since BRD4 is involved in cell cycle arrest (Yang et al., 2008), we examined its impact on the proliferation of myogenic cells using a BrdU incorporation assay that allows for the visualization of cells in the S-phase. Cells were transfected with small interfering RNA targeting *Brd4* (siBrd4) or with scrambled siRNA as a control (siCtrl). The knockdown efficiency of *Brd4* was confirmed by western blot (Figure 2A). Loss of BRD4 reduced BrdU incorporation compared to scrambled controls (Figure 2B). To validate these results, we inhibited BRD4 activity with the chemical inhibitor JQ1 (Filippakopoulos et al., 2010; Devaiah et al., 2016). Similar to the knockdown of *Brd4*, there was a significant reduction in BrdU-positive cells in JQ1-treated cells (Figure 2C). Consistently, knockdown of *Brd4* also reduced proliferation in primary mouse myoblasts (Supplementary Figures S1A,B).

Next, to determine the effects on differentiation, control and siBrd4 cells were maintained in differentiation media for 2 days. Myotube formation was assessed using myosin heavy chain (MHC) immunofluorescence to visualize differentiated cells. The myogenic index assessed by the percentage of MHC^+^ cells was significantly reduced in cells with *Brd4* knockdown (Figure 2D). Expectedly, a dramatic decrease in the myogenic index was also observed in JQ1-treated cells, demonstrating that BRD4 activity is required for myogenesis (Figure 2E). Consistent with the impairment of myogenic differentiation upon *Brd4* depletion, the expression of *Myog* and *Myh1* was reduced in *Brd4* knockdown cells and JQ1-treated cells (Figures 2F,G). The protein levels of MyoG and TnT were also decreased in *Brd4*-knockdown cells and JQ1-treated cells (Figures 2H,I). Similar results on proliferation and differentiation were seen with stable knockdown of *Brd4* (Supplementary Figures S2A–D) indicating that the effects seen with *Brd4* siRNA were not due to off-target effects.

To further confirm that BRD4 is sufficient to enhance myogenesis, we performed transient BRD4 overexpression. Overexpression of BRD4 was confirmed by western blot (Supplementary Figures S2E). A higher number of MHC^+^ cells was apparent in BRD4-overexpressing cells along with elevated levels of MyoG and TnT (Supplementary Figures S2E–G).

### Gene expression analysis in bromodomain-containing protein 4-depleted cells

**FIGURE 3:**
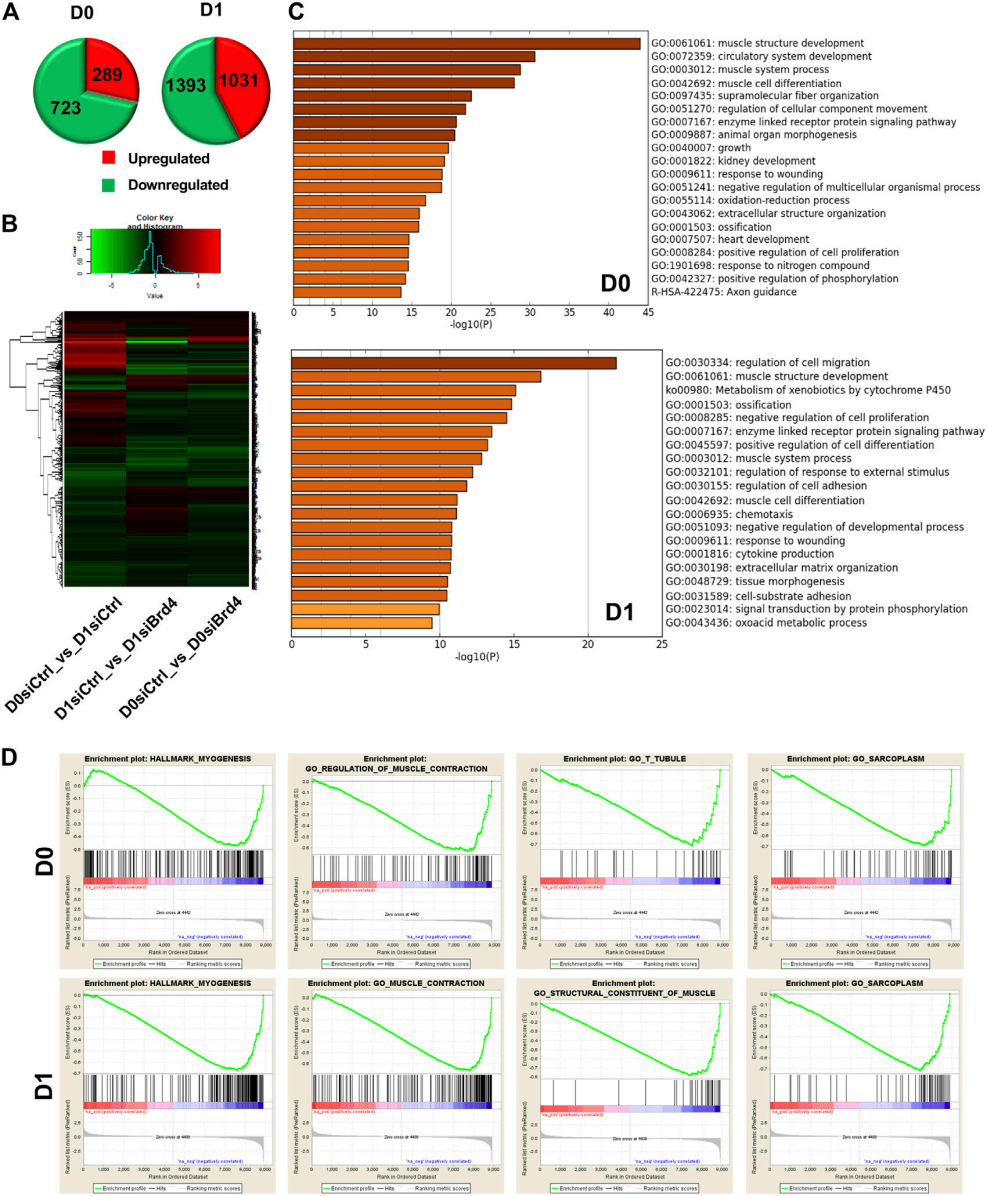
Global gene expression analysis in *Brd4* knockdown myoblasts. **(A)** RNA-seq analysis was performed with three biological replicates of siCtrl and siBrd4 C2C12 cells in proliferating (D0) and differentiating conditions (D1). Pie charts show the total number of significantly upregulated (red) and downregulated (green) genes in siBrd4 cells compared to scrambled control cells at D0 and D1. Differential gene expression was determined by comparing the mean of triplicates. **(B)** A heatmap was generated by listing significantly changed genes upon differentiation (D0 siCtrl vs. D1 siCtrl) and the changes in those genes upon knockdown of *Brd4* at D1 and D0 as indicated. **(C)** Gene ontology (GO) analysis of all significantly altered genes revealed key biological categories and pathways associated with *Brd4* knockdown in D0 and D1 cells. **(D)** Graphical representation of myogenesis and muscle-related pathways regulated by siBrd4 from Gene Set Enrichment analysis (GSEA) at D0 (upper panel) and D1 (lower panel) (siBrd4 compared to siCtrl, FDR q-value ≤ 0.05).

To investigate the mechanisms underlying BRD4-mediated myogenesis, we performed RNA-seq of control and siBrd4 cells. RNA samples from proliferating myoblasts at day 0 (D0) and upon induction of differentiation (D1) were collected in triplicate. In total, 289 genes were found to be significantly upregulated and 723 genes were significantly downregulated in siBrd4 cells at D0, while 1,031 genes were found to be significantly upregulated and 1,393 genes were significantly downregulated at D1 (Figure 3A). Consistent with the role of BRD4 in activating transcription, a number of genes were downregulated in *Brd4* knockdown cells. Additionally, genes that were upregulated upon differentiation (scramble D0 vs. scramble D1) were generally downregulated in *Brd4* knockdown cells, while genes that were downregulated upon differentiation were upregulated in *Brd4* knockdown cells, indicating that BRD4 may control those genes directly (Figure 3B). Gene ontology (GO) analysis further revealed that muscle structure development and muscle cell differentiation were the top regulated biological categories in *Brd4* knockdown cells, suggesting that BRD4 plays important roles in myogenesis (Figure 3C). We also performed Gene Set Enrichment Analysis (GSEA) and found that muscle differentiation (myogenesis) and muscle-related functions (muscle contraction, T-tubule, constituent of muscle and sarcoplasm) were all downregulated in siBrd4 cells (Figure 3D), suggesting that BRD4 may directly activate myogenic gene expression.

### Bromodomain-containing protein 4 directly regulates myogenic genes

**FIGURE 4:**
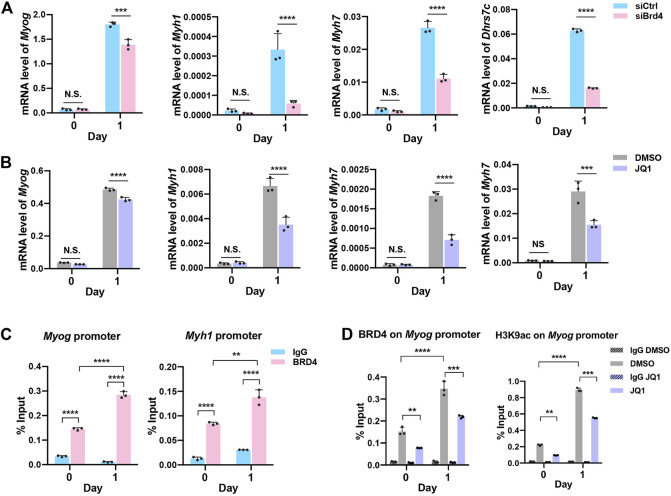
BRD4 regulates the expression of genes involved in myogenesis. **(A,B)** Validation of the *Myog*, *Myh1*, *Myh7,* and *Dhrs7c* expression in siCtrl and siBrd4 cells **(A)** and in JQ1 treated cells **(B)** via qRT-PCR (*n* = 3). **(C)** ChIP assays were performed with an anti-BRD4 antibody at the *Myog* and *Myh1* promoters in proliferating (D0) and differentiating (D1) C2C12 cells. IgG antibody was used as the control (*n* = 3). **(D)** ChIP assays were performed with anti-BRD4 and anti-H3K9ac at the *Myog* promoter in D0 and D1 cells treated with or without JQ1 treatment. Error bars indicate the mean ± SD. (**p < 0.01, ***p < 0.001, ****p < 0.0001, two-way ANOVA with Sidak’s multiple comparisons test).

To validate the RNA-seq results, we performed qRT-PCR analysis of myogenic genes (*Myog*, *Myh1,* and *Myh7*) as well as *Dhrs7c,* which are involved in the regulation of myogenesis. Consistent with our earlier results, a significant reduction in myogenic genes was observed in siBrd4 cells, especially upon induction of differentiation at D1 (Figure 4A). Dehydrogenase/reductase member 7c (Dhrs7c), a member of the short-chain dehydrogenase/reductase (SDR) family, is required for the maintenance of intracellular Ca^2+^ homeostasis, and loss of Dhrs7c affects myotube morphology (Lu et al., 2012; Treves et al., 2012; Arai et al., 2017). Consistent with an earlier study (Arai et al., 2017), we found upregulation of *Dhrs7c* during differentiation (Figure 4A). Upon *Brd4* knockdown, there was a dramatic decrease in *Dhrs7c* mRNA expression in D1 cells (Figure 4A). We also checked the genes in JQ1-treated cells. The mRNA levels of *Myog*, *Myh1,* and *Myh7* as well as *Dhrs7c* were downregulated upon JQ1 treatment (Figure 4B). To test whether these are direct BRD4 targets, we treated cells with JQ1 and performed ChIP-PCR assays on the *Myog* promoter. An increase in BRD4 occupancy and H3K9ac activation marks was apparent at D1 relative to D0 (Figures 4C,D). In JQ1-treated cells, both BRD4 occupancy and H3K9ac enrichment were reduced, indicating that BRD4 directly regulates the expression of *Myog* (Figure 4D).

### Bromodomain-containing protein 4 function in myogenesis is antagonized by G9a

**FIGURE 5:**
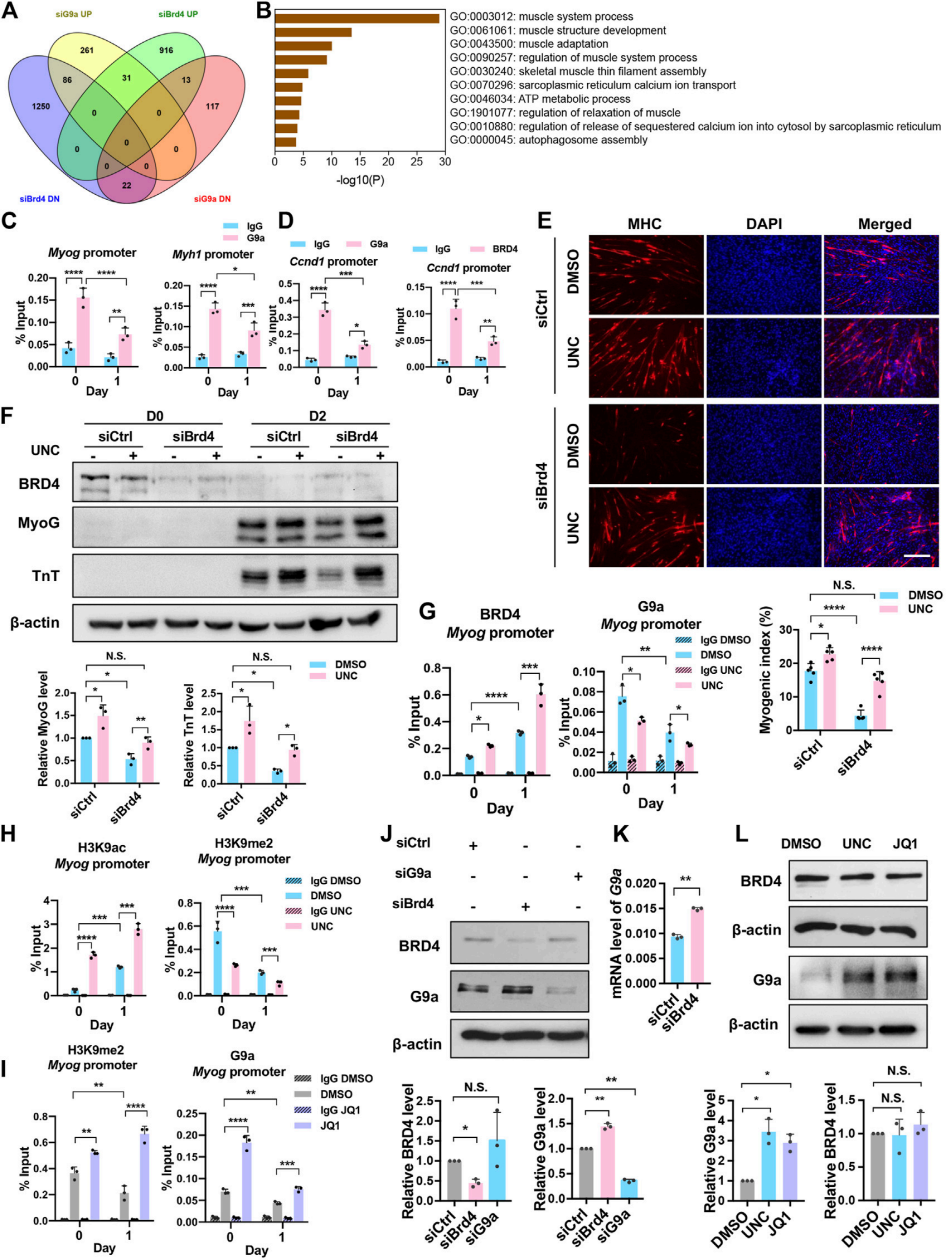
BRD4 inhibits G9a to regulate myogenic differentiation. **(A)** Venn diagram showing the significantly upregulated (UP) and downregulated (DN) genes from the overlap of BRD4 knockdown RNA-Seq and G9a knockdown microarray analysis. **(B)** Gene Ontology (GO) analysis of genes that were downregulated in *Brd4* knockdown cells and upregulated in G9a knockdown cells. **(C)** ChIP assays were performed with anti-G9a antibody at the *Myog* and *Myh1* promoters in D0 and D1 cells. **(D)** ChIP assays were performed with anti-G9a and anti-BRD4 antibodies at the *Ccnd1* promoter in D0 and D1 cells. **(E)** siCtrl or siBrd4 were treated with or without 500 nM UNC0642 for 24 h and collected for D0 or shifted to differentiation medium for 2 days with or without UNC0642. MHC staining was performed. Scale bar, 100 µm. The bar graph below shows the myogenic index (*n* = 5). **(F)** C2C12 cells were treated as in **(E)**, and MyoG and TnT expression was analysed by western blotting. The signals for MyoG and TnT were normalized to β-actin and the relative expression in control and siBrd4 cells is presented in the bar graphs below (*n* = 3). **(G)** ChIP assays were performed with anti-BRD4 and anti-G9a antibodies at the *Myog* promoter in D0 and D1 cells treated with or without UNC0642. **(H)** ChIP assays were performed with anti-H3K9ac and anti-H3K9me2 antibodies at the *Myog* promoter in D0 and D1 cells treated with or without UNC0642. **(I)** ChIP assays were performed with anti-G9a and anti-H3K9me2 antibodies at the *Myog* promoter in D0 and D1 cells in the absence or presence of JQ1. **(J)** C2C12 cells were transfected with siCtrl, siBrd4 or siG9a for 48 h. BRD4 and G9a expression was analysed by western blotting (*n* = 3). The signals for BRD4 and G9a were normalized to β-actin and are quantified in the bar graph below. **(K)** C2C12 cells were transfected with either siCtrl or siBrd4 for 48 h G9a mRNA level was analysed by qRT-PCR (*n* = 3). **(L)** C2C12 cells were treated with either 500 nM UNC0642 or 50 nM JQ1. DMSO was added as a control. G9a and BRD4 expression was analysed by western blotting. The signals for BRD4 and G9a were normalized to β-actin and are quantified in the bar graph below (*n* = 3). Error bars indicate the mean ± SD. (*p < 0.05, **p < 0.01, ***p < 0.001, ****p < 0.0001. Two-way ANOVA with Sidak’s multiple comparisons test).

Our previous studies have shown that the lysine methyltransferase G9a inhibits myogenesis (Ling et al., 2012). Given the opposing effects of G9a and BRD4 during myogenesis, we examined whether BRD4 positively regulates myogenesis by inhibiting G9a. We therefore integrated the siBrd4 RNA-seq data with microarray data from G9a knockdown cells (Rao et al., 2016). Interestingly, 86 genes that were upregulated in siG9a cells were downregulated in siBrd4 cells (Figure 5A). GO analysis of the 86 genes *via* Metascape revealed that these were mainly involved in muscle system and muscle development (Figure 5B) (Zhou et al., 2019). We also checked the genes that were similarly regulated by G9a and BRD4. The 31 genes upregulated upon both G9a and BRD4 loss did not show any enrichment of muscle development terms. Although the 22 downregulated genes showed some enrichment of muscle contraction, it was not as strong as the inversely correlated 86 genes. All the above results suggested that G9a and BRD4 have opposing roles in the regulation of myogenic genes. We then performed G9a and BRD4 ChIP to determine their temporal occupancy at the *Myog, Myh1,* and *Ccnd1* promoters. As shown in Figures 5C,D, an enrichment of G9a was seen on the *Myog, Myh1,* and *Ccnd1* gene promoters compared to the IgG control at D0. G9a occupancy on the *Myog and Myh1* promoters decreased, while the occupancy of BRD4 increased upon differentiation at D1 (Figures 4C, 5C), indicating that both G9a and BRD4 directly regulate myogenic genes. Interestingly, the occupancy of both enzymes on the *Ccnd1* promoter decreased upon differentiation (Figure 5D).

We next tested whether G9a and BRD4 independently regulate the same genes or whether G9a is involved in BRD4-mediated myogenesis. To address this, we examined whether the differentiation block in siBrd4 cells is due to G9a by treating siBrd4 cells with the G9a inhibitor UNC0642. Interestingly, upon treatment with UNC0642, the differentiation defect caused by *Brd4* knockdown was rescued as evidenced by MHC staining, the myogenic index and the expression of MyoG and TnT (Figures 5E,F). To further validate these results, we tested BRD4 occupancy at the *Myog* promoter in control and UNC0642-treated cells. BRD4 enrichment was dramatically increased in UNC0642-treated cells at D1 (Figure 5G). Correspondingly, an increase in H3K9ac and a decrease in H3K9me2 was observed, demonstrating that BRD4 function is negatively regulated by G9a (Figure 5H). To further test the antagonism between G9a and BRD4 occupancy at myogenic gene promoters, we treated cells with JQ1 and performed ChIP-PCR assays at the *Myog* promoter. In JQ1-treated cells, both BRD4 occupancy and H3K9ac enrichment were reduced (Figure 4D). In contrast, G9a enrichment and H3K9me2 marks were increased at both D0 and D1 upon inhibition of BRD4 (Figure 5I).

To examine the basis of antagonism between BRD4 and G9a, we tested whether they regulate each other’s expression. In siBrd4 and JQ1-treated cells, a modest, albeit significant increase in G9a was observed (Figures 5J,K). On the other hand, no significant change in BRD4 levels was observed in siG9a cells or UNC-treated cells (Figures 5J,L).

## Discussion

In this study, we provide evidence of an interplay between BRD4 and G9a that controls the expression of skeletal muscle differentiation genes. Several lines of evidence support crosstalk between BRD4 and G9a in myogenesis. First, an overlap of the transcriptomic data from BRD4-and G9a-depleted cells identified a subset of differentiation genes that are downregulated by loss of BRD4 and upregulated by loss of G9a. Second, upon induction of differentiation, endogenous BRD4 enrichment is increased, whereas G9a enrichment is reduced at these myogenic gene promoters. Third, treatment with the BRD4 inhibitor JQ1 resulted in increased G9a protein levels as well as occupancy of the G9a and H3K9me2 marks. Conversely, inhibition of G9a activity with UNC0642 enhances BRD4 occupancy and H3K9ac marks at myogenic promoters. Fourth, inhibition of G9a activity functionally rescued myogenic differentiation, demonstrating that BRD4 positively regulates skeletal myogenesis *via* suppression of G9a activity.

Histones are subject to various post-translational modifications. These modifications are dynamically controlled to allow the precise regulation of gene expression. Several studies have demonstrated crosstalk between histone PTMs such that one modification activates chromatin modifying complexes, and in turn, generates a different modification (Suganuma and Workman, 2008; Janssen and Lorincz, 2022). In addition, opposing epigenetic marks such as methylation and acetylation can be deposited on the same residue. For instance, H3K9 can be targeted for methylation and acetylation that have inverse outcomes resulting in gene repression or activation. Thus, one modification can act as a barrier for another presumably by antagonism between the effector enzymes.

Our findings are consistent with a previous report which showed that BRD4 promotes cell cycle progression as well as myogenic differentiation (Roberts et al., 2017). Interestingly, this study showed that the function of BRD4 in cell cycle progression is uncoupled from its role in differentiation. On the other hand, BRD3 was found to block differentiation indicating opposing roles of BRD3 and BRD4 in myogenesis. The mechanisms underlying the antagonism between BRD3 and BRD4 need further investigation. Our study expands on these findings by demonstrating that BRD4 promotes differentiation by suppressing G9a and unveil an interplay between BRD4 and G9a to fine tune the expression of myogenic genes. It is intriguing that both BRD4 and G9a are expressed in myoblasts, and both are downregulated during differentiation. They have a similar function in myoblast proliferation and promote cell cycle progression (Yang et al., 2008; Rao et al., 2016; Srinivasan et al., 2019). In myoblasts, knockdown or inhibition of BRD4 *via* JQ1 reduced proliferation, and *Ccnd1* mRNA levels were decreased. Moreover, several reports have shown that BRD4 promotes cancer cell proliferation and that inhibition of BRD4 leads to cancer cell death (White et al., 2019; Tan et al., 2020). In the context of hematological and solid malignancies, BET inhibitors (BETis) are now considered as one of the most promising therapeutic strategies. Similarly, G9a enhances myoblast proliferation by enhancing E2F1 target gene expression and is upregulated in many cancers (Rao et al., 2016; Chae et al., 2019; Segovia et al., 2019; Pal et al., 2020; Souza et al., 2021). Nevertheless, BRD4 and G9a have opposing effects on myogenic differentiation, and elevated G9a protein level/activity appears to underlie the differentiation block of differentiation in *Brd4*-depleted cells. Our data demonstrate that G9a expression is modestly elevated in the absence of BRD4. It is plausible that additional mechanisms contribute to the antagonistic relationship. Previous studies have shown that BRD4 interacts with G9a (Wu et al., 2013). Moreover, BRD4 inhibits autophagy in human pancreatic ductal adenocarcinoma cells by associating with G9a (Sakamaki et al., 2017; Sakamaki and Ryan, 2017; Shi et al., 2021). As G9a levels decline during myogenic differentiation, H3K9me2 marks at myogenic gene promoters would be reduced (Ling et al., 2012), that allows KATs to deposit activating acetylation marks which are read by BRD4 to promote myogenesis. Despite its pro-myogenic role, BRD4 levels decline at late stages of differentiation. This finding is consistent with a previous report showing that BRD4 expression is decreased under growth inhibition conditions (Dey et al., 2000). Nevertheless, the expression pattern suggests that sustained BRD4 presence is not required for its pro-differentiation effects. It would be interesting to determine whether the crosstalk between BRD4 and G9a in myoblast proliferation and differentiation occurs through the formation of distinct protein complexes.

## Data Availability

The datasets presented in this study can be found in online repositories. The names of the repository/repositories and accession number(s) can be found below: Gene Expression Omnibus accession number: GSE141777.
