# Supplementary material for: An interplay between BRD4 and G9a regulates skeletal myogenesis
Source: Front Cell Dev Biol. 2022 Sep 7;10:978931. doi: 10.3389/fcell.2022.978931 (PMC9489841; doi:10.3389/fcell.2022.978931)
Supplement: Supplementary file 1 [file DataSheet1.docx]

Supplementary Material


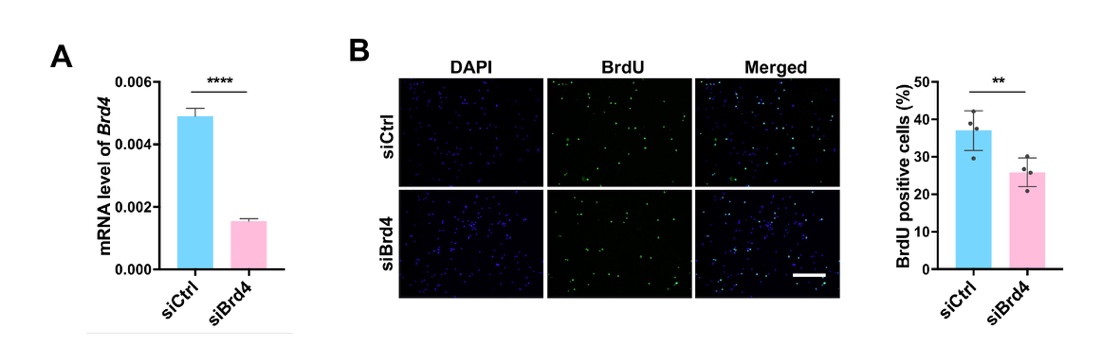


**Supplementary Figure 1.** *Brd4* knockdown reduces cell proliferation. (A) Primary mouse myoblasts were transfected with either siCtrl or siBrd4 for 48 h. siRNA-mediated *Brd4* knockdown efficiency of same bench of panel B was assessed by q-PCR. (B) Primary mouse myoblasts were treated as in (A) and then pulsed with BrdU and stained with anti-BrdU antibody (left panel). The percentage of BrdU-positive cells was quantified (right panel), n = 4 independent experiments. Error bars indicate the mean ± SD (** *p* < 0.01, *****p* < 0.0001, two-tailed t tests were performed).


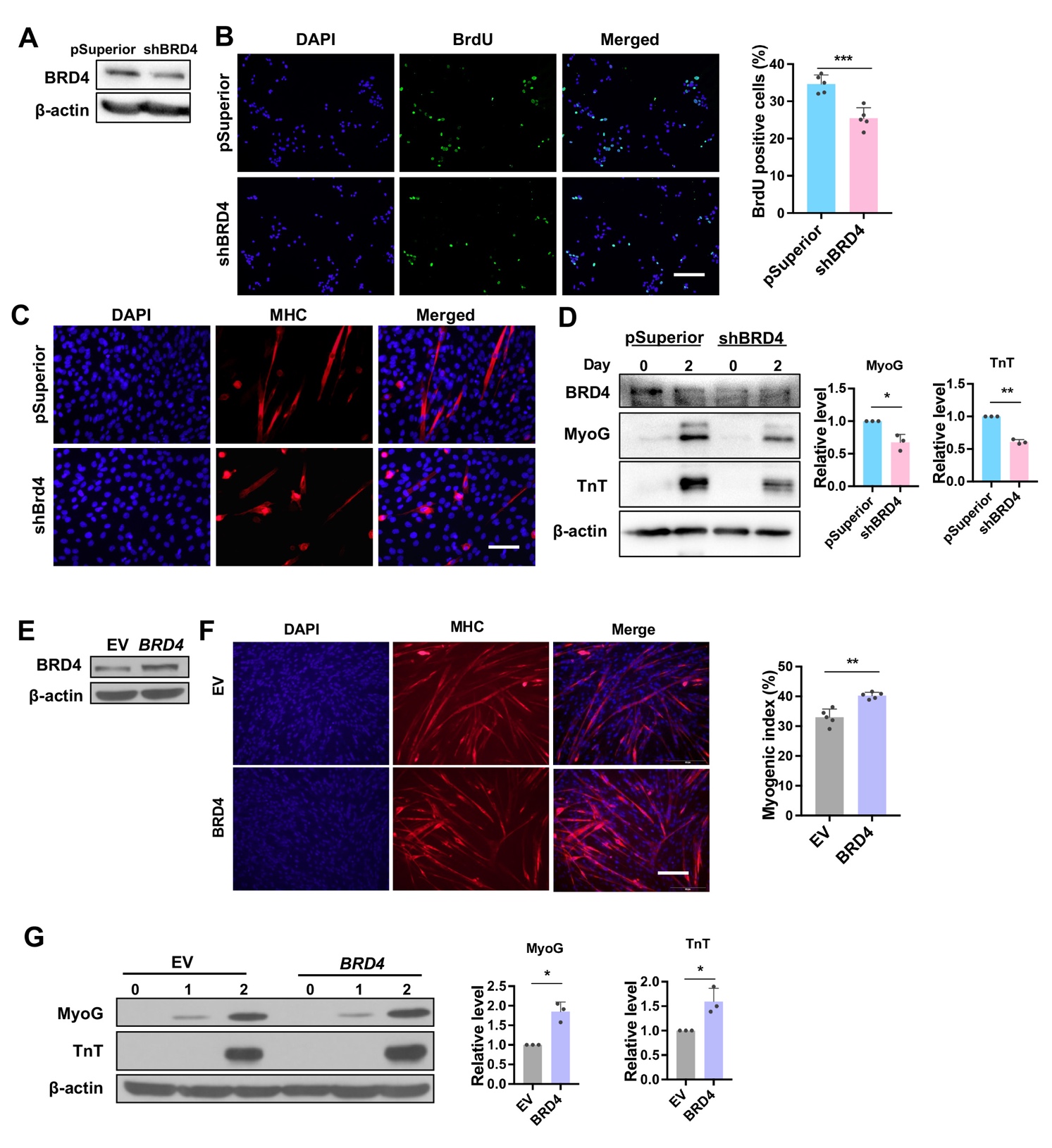


**Supplementary Figure 2.** BRD4 promotes myogenesis. (A) C2C12 cells were transfected with pSuperior and shBRD4 plasmids (for human BRD4 gene) to create *Brd4* stable knockdown cells. The knockdown efficiency was assessed by western blotting. (B) *Brd4* stable knockdown cells were pulsed with BrdU and stained with anti-BrdU antibody (left panel). The percentage of BrdU-positive cells was quantified by counting at least 1000 cells (right panel), n = 5 independent experiments. (C) pSuperior cells or *Brd4* stable knockdown C2C12 cells were differentiated for two days, and MHC staining was performed, n = 3 independent experiments. (D) pSuperior cells or *Brd4* stable knockdown C2C12 cells were treated as in (C), and myogenic markers were checked via western blotting. (E) C2C12 cells were transfected with either empty vector (EV, pcDNA) or pcDNA-BRD4 plasmid to get transient BRD4overexpression C2C12 cells. The BRD4 expression efficiency was assessed by western blotting. (F) EV transfected or BRD4-overexpression C2C12 cells were differentiated for two days, and MHC staining was performed, n = 5 independent experiments. (G) EV transfected or BRD4-overexpression C2C12 cells were differentiated for two days, and myogenic markers were checked via western blotting, n = 3 independent experiments. Error bars indicate the mean ± SD (* *p* < 0.05, ** *p* < 0.01, ****p* < 0.001, two-tailed t tests were performed).

.
